# Underweight and overweight men have greater exercise-induced dyspnoea than normal weight men

**DOI:** 10.3109/03009734.2012.714416

**Published:** 2012-10-30

**Authors:** Mirza m. F. Subhan, Syed a. Ali, Syed S. I. Bokhari, Mohammed n. Khan, Hakimuddin r. Ahmad

**Affiliations:** ^1^Department of Physiology, College of Medicine and Medical Sciences, Arabian Gulf University, Kingdom of Bahrain; ^2^Department of Biological & Biomedical Sciences, Faculty of Health Sciences, The Aga Khan University, Karachi, Pakistan; ^3^Faculty of Health Sciences, Jinnah Medical College, Karachi, Pakistan

**Keywords:** Body mass index, dyspnoea, exercise test, respiratory function tests, respiratory muscles

## Abstract

**Introduction.:**

Persons with high or low body mass index (BMI), involved in clinical or mechanistic trials involving exercise testing, might estimate dyspnoea differently from persons with a normal BMI.

**Aims.:**

Our objective was to investigate the relationship between BMI and dyspnoea during exercise in normal subjects with varying BMI.

**Material and methods.:**

A total of 37 subjects undertook progressive exercise testing. Subjects were divided into three groups: underweight (UW), normal weight (NW), and overweight (OW). Dyspnoea was estimated using the visual analogue scale (VAS). Spirometry, maximum voluntary ventilation (MVV), and respiratory muscle strength (RMS) were measured.

**Results and discussion.:**

The intercept of the VAS/ventilation relationship was significantly higher in NW subjects compared to UW (*P* = 0.029) and OW subjects (*P* = 0.040). Relative to the OW group, FVC (*P* = 0.020), FEV_1_ (*P* = 0.024), MVV (*P* = 0.019), and RMS (*P* = 0.003) were significantly decreased in the UW group. The greater levels of dyspnoea in UW subjects could possibly be due to decreased RMS. Healthy persons should aim to achieve an optimum BMI range to have the lowest exercise-induced dyspnoea.

## Introduction

Dyspnoea is a common symptom in many respiratory disorders, and it is also seen in healthy subjects during exercise (1). Increasing grades of dyspnoea in patients have been associated with greater impairment in quality of life (2). After adjusting for age, smoking history, and occupation, dyspnoea has also been shown to be predictive of mortality in elderly people (3). Assessments of dyspnoea during exercise have been made in healthy subjects (4), in chronic obstructive pulmonary disease (COPD) patients to study the benefits of inspiratory muscle training (5), and in COPD patients investigating the effect of therapeutic agents (6).

Abnormalities in body mass index (BMI) have been shown to affect exercise-induced dyspnoea in obese cardiac patients (7). In this study, obese patients were more breathless compared to overweight (OW) or normal weight (NW) patients, during a progressive exercise test. With regard to underweight (UW) and OW subjects and their estimations of exercise-induced dyspnoea, no study could be found in the literature. Previous studies have also shown that abnormal BMI can be related to pulmonary complications. For example, there is a relationship between low BMI and an increased risk of developing COPD (8). Similarly, having a low or high BMI has been associated with a greater prevalence of asthma (9).

Therefore, is it possible that UW or OW persons, if involved in dyspnoea testing, for clinical trials or for mechanistic studies of the effects of dyspnogenic agents, might estimate dyspnoea differently relative to NW persons? A recent breathlessness survey has suggested that future clinical studies for breathlessness should consider stratification by BMI (10). The purpose of the present study was to determine whether a relationship exists between BMI and the perception of dyspnoea during exercise in UW, NW, and OW subjects.

## Material and methods

This was a prospective study, in which healthy subjects underwent exercise and lung function testing. Subjects undertook progressive symptom-limited sub-maximal bicycle ergometry during the course of which dyspnoea was estimated each minute using a visual analogue scale (VAS). Exercise testing was conducted twice, to familiarize the subjects with the procedure; the time period between tests was 1 week.

Thirty-seven healthy subjects (all male) were studied. All were volunteers and were recruited from the staff and students of the Aga Khan University (AKU), Karachi, Pakistan, and all were Pakistani. All were non-smokers, with no history of cardiopulmonary or other chronic disease. Prior to participation, the subjects completed a pre-exercise testing screening questionnaire and a resting electrocardiogram (ECG). Their lung function was assessed by spirometry. Based on their BMI, subjects were divided into three groups: underweight (*n* = 13; < 18.5 kg/m^2^), normal weight (*n* = 12; 18.5–24.9 kg/m^2^), and overweight (*n* = 12; > 25.0 kg/m^2^). Mean BMI (± SD) was 22.6 ± 5.2 kg/m^2^, range 14.5–35.5 kg/m^2^. Mean age (± SD) was 21.7 ± 4.5 years, range 18–37 years. All subjects were sedentary individuals with minimal levels of habitual activity.

Experiments were performed at approximately the same time of the day, at least 2 hours after a light meal. Informed consent was obtained from the subjects, and the study was approved by the AKU Human Subject Protection Committee on 15 June 2001 and performed according to the Declaration of Helsinki. Height and weight were taken on a combined stadiometer/weighing scale (Seca, Hamburg, Germany), with subjects wearing light indoor clothing and no shoes.

The exercise tests were conducted on an electrically braked bicycle ergometer (Tunturi, Piispanristi, Finland). The subject breathed through a valve box, which allowed inspiration from room air. Inspired ventilation (V_I_) and respiratory frequency were measured using a rotating vane anemometer and ventilation monitor (PK Morgan Ltd, Chatham, Kent, UK). The ECG (Datascope, Mahwah, New Jersey, USA) was monitored throughout the test. Heart rate and oxygen saturation were monitored throughout the experiment using a pulse oximeter (Ohmeda, Madison, Wisconsin, USA), attached via a finger probe. The pulse oximeter and ventilation monitor were connected to a personal computer which processed and recorded data every 15 s. Once attached to the equipment, the subject was given a few min to become acquainted to the apparatus. Expired gases were not measured.

The VAS was used to quantify dyspnoea intensity, and it was drawn as a 100 mm linear line on a card, the extremes of which were labelled ‘not at all breathless' (0 mm) and ‘extremely breathless' (100 mm). The subject was given approximately 5 s to mark his response in pencil on the card which was held before him at the end of each minute of the exercise procedure. A separate card was used every min.

Before the start of each experiment the term ‘breathlessness' was defined to the subjects as a sense of breathing discomfort, a feeling that breathing was not sufficient for the needs they thought they had. It was emphasized that they should not confuse breathlessness with other sensations associated with exercise, such as leg fatigue. During both exercise tests, subjects were asked to use the same criteria to estimate their dyspnoea.

Data were recorded for 1 min at rest, and then the subject started unloaded pedalling, for 1 min, at a frequency of between 50–60 revolutions per min. Thereafter, the work rate was increased 20 watts per min until a symptom-limited sub-maximum was reached. The test was stopped if the subject indicated chest pain, showed pallor, severe desaturation, a heart rate within 10 beats min^-1^ of the maximum predicted for that individual (4), indicated greater than 90 mm on the VAS, or if there were any significant ECG abnormalities.

Respiratory muscle strength (RMS) was calculated by the addition of the maximum static inspiratory (P_Imax_) and expiratory (P_Emax_) mouth pressures divided by two (11). P_Imax_ and P_Emax_ were measured at residual volume and total lung capacity, respectively (Med Graphic Profiler, Pulmonary Diagnostic System; Medical Graphics Corp, St Paul, Minnesota, USA). Tests were conducted according to American Thoracic Society (ATS)/European Respiratory Society recommendations (12). Three measurements were taken, and the highest values were chosen, irrespective of the test. This test could not be performed on three subjects, as they were either on leave or had examinations. Although P_Imax_ values are negative, for clarity they were reported as positive numbers, as has been done previously (13).

Spirometry and maximum voluntary ventilation (MVV) were performed on a Compact Vitalograph electronic spirometer (Vitalograph, Maids Moreton, Buckingham, UK). The apparatus was calibrated daily with a 1 litre calibration syringe. Tests were conducted according to ATS recommendations (14). For spirometry, three to five manoeuvres were performed after adequate rest. The highest lung function values were chosen, irrespective of the test. MVV was measured for 15 s, and the frequency of duration exceeded 80 breaths min^-1^ in all subjects. MVV was performed twice, and the highest value was chosen.

When the subjects' VAS estimation was plotted against V_I_, a linear VAS/V_I_ relationship was observed. Correlating VAS against ventilation has been previously performed (4,15,16). Linear correlation was applied, and from this the *x*-axis intercept (L min^-1^) and the slope (mm min L^-1^) were calculated as done before (4). The *x*-axis intercept will be referred to as the intercept. As the first test was to familiarize subjects, only exercise and breathlessness data from week two were analysed. Heart rate was analysed as the heart rate after the first min of exercise (f_C1min_), heart rate at 60 watts of exercise (f_C60_), and maximum heart rate (f_Cmax_).

For dyspnoea comparisons between groups, one-way ANOVA was used for analysis. Bonferroni's *post hoc* analysis was used for comparisons between specific groups. Stepwise multiple linear regression analysis of the slope and intercept against predictor variables such as age, height, etc. was also made. All spirometric test data were pooled and analysed using repeated measures analysis of covariance (ANCOVA), including age and height and two dummy variables to describe the three groups. All tests were two-tailed, and the level of probability taken as significance was 5% (*P* < 0.05).

## Results

### Lung function

Relative to the OW group, forced vital capacity (FVC) (*P* = 0.020), forced expiratory volume in 1 second (FEV_1_) (*P* = 0.024), and MVV (*P* = 0.019) were significantly decreased in the UW group, while FVC was lower in NW subjects (*P* = 0.028) (Table I). The P_Imax_ (*P* = 0.008), P_Emax_ (*P* = 0.010), and RMS (*P* = 0.003) were also significantly lower for the UW group compared to the OW group. Age, height, FEV_1_/FVC % ratio, and peak expiratory flow (PEF) did not show any significant changes between groups.

**Table I.** Mean (± SD) anthropometric and lung function variables for all subjects in underweight, normal weight, and overweight groups (*n* = 37).

	Underweight (*n* = 13)	Normal (*n* = 12)	Overweight (*n* = 12)	*P* value^a^
Age (years)	21.4 ± 4.2	22.0 ± 4.3	21.7 ± 5.3	0.953
BMI (kg m^-2^)	17.4 ± 1.1	22.3 ± 2.1	28.7 ± 2.9	<0.0001
Height (cm)	174 ± 6	173 ± 4	175 ± 9	0.809
Weight (kg)	52 ± 5	66 ± 8	89 ± 15	<0.0001
FVC (L)	4.13 ± 0.65	4.15 ± 0.61	5.10 ± 1.18	0.010
FEV_1_ (L)	3.32 ± 0.60	3.48 ± 0.61	4.12 ± 0.88	0.020
FEV_1_/FVC % ratio (%)	79.4 ± 7.8	83.7 ± 6.3	80.9 ± 3.1	0.226
PEF (L min^-1^)	511.8 ± 123.5	556.9 ± 103.5	588.0 ± 103.4	0.238
MVV (L min^-1^)	136.2 ± 29.8	143.7 ± 32.0	170.6 ± 41.6	0.048
P_Imax_ (cmH_2_O)^b^	79.8 ± 34.0	106.3 ± 36.8	144.9 ± 66.4	0.010
P_Emax_ (cmH_2_O)^b^	80.4 ± 24.3	107.3 ± 19.6	111.9 ± 25.7	0.006
RMS (cmH_2_O)^b^	80.1 ± 23.5	106.9 ± 25.6	128.5 ± 43.4	0.004

^a^Difference between three groups.

^b^ *n* = 12, 12, and 10 per group, respectively. BMI = body mass index; FEV_1_ = forced expiratory volume in 1 s; FEV_1_/FVC % ratio = ratio of FEV_1_ to FVC; FVC = forced vital capacity; MVV = maximum voluntary ventilation; PEF = peak expiratory flow; P_Emax_ = maximum static expiratory mouth pressure; P_Imax_ = maximum static inspiratory mouth pressure; RMS = respiratory muscle strength.

Results for spirometry and MVV are presented as actual values and not corrected for age and height. One reason for this was that there were no significant differences in age and height between groups, and secondly there are no standardized values for the Pakistani population. However, we did analyse the data as a percentage of Caucasian references values, which took into account age and height, and found no disparity between the two methods of analysis in terms of significantly different variables. Using ANCOVA, no significant difference was found in the repeated spirometric data between the three BMI groups, taking into account both subject height and age, FVC (*P* = 0.71), FEV_1_ (*P* = 0.76), FEV_1_/FVC % ratio (*P* = 0.93), or PEF (*P* = 0.93).

### Exercise-induced dyspnoea

The relationship between VAS and V_I_ was significant for all subjects (*P* < 0.05) and effectively linear for all groups. The mean (± SD) *r^2^* (coefficient of determination), for all experiments was 0.959 ± 0.030 (range 0.882–0.999); *r^2^* was significantly different between groups (Table II). There was a wide range of values for the slope (0.6–12.1 mm min L^-1^) and intercept (3.4–42.0 L min^-1^) of the VAS/V_I_ relationship in all subjects.

**Table II.** Comparison of mean (± SD) indices of the breathlessness/ventilation relationship and exercise variables between underweight, normal weight, and overweight groups (*n* = 37).

	Underweight (*n* = 13)	Normal (*n* = 12)	Overweight (*n* = 12)	*P* value^a^
*r^2^* value	0.94 ± 0.03	0.97 ± 0.02	0.96 ± 0.03	<0.050
VAS/V_I_ slope (mm min L^-1^)	2.73 ± 2.99	2.05 ± 1.40	1.96 ± 1.04	0.575
VAS/V_I_ intercept (L min^-1^)	16.1 ± 5.84	22.7 ± 7.54	17.1 ± 3.96	0.022
W_max_ (W)	113.9 ± 25.0	120.0 ± 29.5	126.7 ± 27.4	0.510
W_max_ per kg (W/kg)	2.17 ± 0.53	1.80 ± 0.36	1.46 ± 0.39	0.001
V_Imax_ (L min^-1^)	48.0 ± 12.0	45.3 ± 11.2	48.6 ± 8.9	0.725
f_C1min_ (beats min^-1^)	91.8 ± 12.9	88.0 ± 13.5	91.9 ± 13.6	0.711
f_C60_ (beats min^-1^)	123.7 ± 15.4	116.8 ± 12.3	113.8 ± 16.3	0.239
f_Cmax_ (beats min^-1^)	153.0 ± 22.0	145.7 ± 18.8	144.7 ± 19.1	0.533

^a^Difference between three groups.

f_C1min_ = heart rate after 1 min of exercise; f_C60_ = heart rate at 60 watts of exercise; f_Cmax_ = maximum heart rate; *r^2^* = coefficient of determination; VAS/V_I_ = relationship of visual analogue scale against inspired ventilation; V_Imax_ = maximum ventilation; W_max_ = maximum work-load; W_max_ per kg = W_max_ per kg body mass.

**Figure 1.**
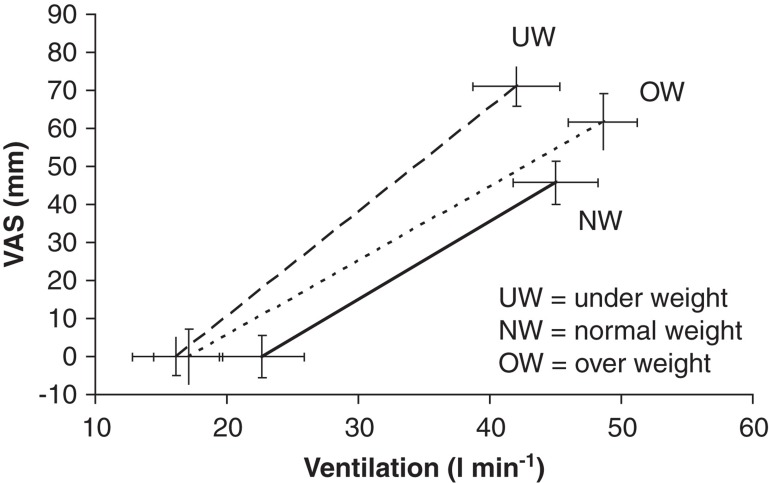
A representation of the mean changes in the VAS/V_I_ relationship for all subjects in the three BMI groups. There were no differences in the slope values, but the NW intercept value was significantly greater relative to values for UW and OW subjects.

The VAS/V_I_ intercept (Table II) in the NW group was significantly higher than in both the UW (*P* = 0.029) and OW groups (*P* = 0.040). UW maximum work-load (W_max_) per kg body mass values were significantly higher than corresponding OW values (*P* = 0.001). There were no statistically significant differences in VAS/V_I_ slope, W_max_, maximum ventilation (V_Imax_), f_C1min_, f_C60_, and f_Cmax_ between the groups tested (Table II). The mean intercept and slope values of the relationships between VAS and inspired ventilation for the three groups have been graphically illustrated (Figure 1).

### Regression analyses

Linear correlations of BMI, lung function, exercise, and dyspnoea parameters were made, combining data from all groups (Table III). BMI had significant positive correlations with RMS, P_Imax_, MVV, FVC, FEV_1_, and PEF and a negative correlation with f_C60_. The P_Imax_ showed positive relationships against FVC and FEV_1_, while it showed a negative correlation with W_max_/body mass. The slope of the VAS/V_I_ relationship was negatively correlated with RMS and *r^2^*. The f_C60_ had significant negative correlations with MVV, P_Imax_, FVC, and FEV_1_.

**Table III.** Linear correlation coefficients for selected correlations, using data from all subjects.

Correlations	*r* value (correlation coefficient)	*P* value
BMI versus RMS	+0.448	0.008
BMI versus MVV	+0.419	0.009
BMI versus FVC	+0.454	0.004
BMI versus PEF	+0.333	0.044
BMI versus f_C60_	–0.339	0.040
P_Imax_ versus FVC	+0.409	0.018
P_Imax_ versus W_max_ per kg	–0.373	0.030
VAS/V_I_ slope versus RMS	–0.377	0.028
VAS/V_I_ slope versus *r^2^*	–0.407	0.012
f_C60_ versus MVV	–0.453	0.005
f_C60_ versus P_Imax_	–0.378	0.027
f_C60_ versus FVC	–0.490	0.002

BMI = body mass index; f_C60_ = heart rate at 60 watts of exercise; FVC = forced vital capacity; MVV = maximum voluntary ventilation; PEF = peak expiratory flow; P_Imax_ = maximum static inspiratory mouth pressure; *r^2^* = coefficient of determination; RMS = respiratory muscle strength; VAS/V_I_ = relationship of visual analogue scale against inspired ventilation; W_max_ per kg = maximum work-load per kg body mass.

Using the stepwise method, significant models emerged for the multiple regression analysis of the VAS/V_I_ slope (*F*_3,31_ = 29.330, *P* < 0.00001, adjusted *r^2^* = 0.714) and VAS/V_I_ intercept (*F*_1,33_ = 218.824, *P* < 0.00001, adjusted *R* square = 0.865). Significant predictor variables for the slope are the FEV_1_/FVC % ratio, V_Imax_, and P_Emax_ (Table IV). For the intercept, the VAS/V_I_ *r^2^* value was the only significant variable. Residuals for the slope and intercept from the regression were plotted against BMI, P_Imax_, and P_Emax_; data points were randomly distributed around zero.

**Table IV.** Multiple regression analysis of the VAS/V_I_ slope and intercept, showing significant predictor variables.

Dependent variable	Independent variable (predictor)	Standardized beta coefficient	*P* value
VAS/V_I_ slope	FEV_1_/FVC % ratio	2.684	<0.00001
	V_Imax_	–1.184	0.004
	P_Emax_	–0.811	0.029
VAS/V_I_ intercept	VAS/V_I_ *r^2^* value	0.932	<0.00001

FEV_1_/FVC % ratio = ratio of FEV_1_ to FVC; P_Emax_ = maximum static expiratory mouth pressure; *r^2^* = coefficient of determination; VAS/V_I_ = relationship of visual analogue scale against inspired ventilation; V_Imax_ = maximum ventilation.

## Discussion

The main finding of our study was that UW and OW subjects, relative to NW subjects, showed greater levels of dyspnoea during exercise, due to a lower VAS/V_I_ intercept, indicating that these subjects estimated their sensations of exercise-induced dyspnoea earlier than NW subjects. We also found that FVC, FEV_1_, and MVV were significantly decreased in the UW group, relative to the OW group; changes in FVC and FEV_1_ are in agreement with a previous report (17). An earlier study showed a positive correlation between BMI with both FVC and FEV_1_ (18). Schachter et al. suggested that an inferior lung function of UW subjects could be due to a decrease in RMS (17). FVC, though not FEV_1_, in our OW subjects was significantly greater relative to NW subjects. In contrast, previous data reported no significant differences in FVC and FEV_1_ between OW and NW subjects (17).

The present study also showed that both RMS and P_Imax_ were significantly lower for the UW group compared to the OW group; both values showed a positive correlation with body mass, in agreement with previous work in healthy subjects (19). Combined P_Emax_ data for UW patients without lung disease and controls have also shown a significant positive correlation with body weight (11).

Diaphragm muscle mass has been significantly correlated to body mass in normal subjects after autopsy (20). This could be a basis for the significant relationship between BMI with RMS and P_Imax_ in the present study. The higher RMS of the OW subjects may be due to the increase in body mass improving their muscle strength (21) and vice versa in UW subjects.

P_Imax_ and several spirometric variables were significantly negatively correlated to f_C60_, indicative that persons with lower exercise heart rates had better lung function. This is supported by work showing an association between physical activity and lung function (22).

At any given level of ventilation, UW and OW subjects were significantly more breathless than NW subjects. NW subjects were the least breathless. The relationship between dyspnoea and ventilation was linear, as shown by the *r^2^* value, and had a large inter-subject variation, consistent with previous work (1,4).

It is interesting to note that although our VAS/V_I_ slope was not significantly affected by changes in BMI, it had a significant negative correlation with RMS and P_Emax_, indicating that a decrease in muscle strength was associated with greater exercise dyspnoea.

Obesity increases dyspnoea (17,23), and reasons for this include an increase in the work of breathing, due to an increased elastic resistance to distension (24), increased respiratory or nasopharyngeal resistance (25), increased pulmonary venous pressures (26), and greater respiratory work (27). A greater work of breathing suggests a decreased ventilatory reserve, which predisposes these persons to respiratory failure and developing dyspnoea. Weight reduction in the obese, by diet and exercise or surgery, can improve dyspnoea (26). Most of our OW subjects were not obese, and they were unlikely to possess the respiratory and haemodynamic alterations mentioned above, especially since their lung function was better than that of the NW subjects in our study.

The current hypothesis for the genesis of dyspnoea is that output of the central motor command from the brainstem to the respiratory muscles is also projected to the sensory cortex (1). Human neuroimaging has shown that the insula is essential for dyspnoea perception (28). It has been suggested that the sensory cortex interacts with the insular cortex to modulate the dyspnoea intercept (4). It could be possible that our UW subjects, who had weaker respiratory muscles, had a greater efferent output from the brainstem, thereby increasing their dyspnoea. In OW subjects, the excess body mass could result in a greater work of breathing and more afferent input to the brainstem, which would again result in a greater efferent output from the brainstem. It is unclear as to why the threshold (intercept) and not the gain (slope) are affected by BMI.

Limitations of the present study are that a larger sample size for each group could have shown more definitive results. The study participants were all men, therefore extrapolating the results to women cannot be done. In the present study we have speculated that changes in the VAS/V_I_ intercept in UW subjects were most likely due to changes in RMS. An alternative explanation could be that this group was less physically active and conditioned than the other group; however, when exercise heart rates were compared, there were no significant differences. Another limitation of this study is that we could not distinguish whether changes in BMI were due to changes in lean tissue mass or fat mass. In addition, most of our subjects were sedentary individuals with poor levels of physical fitness. This might limit generalizing the results with a population having normal or good levels of physical fitness.

The OW group had eight overweight and five obese subjects; their mean BMI was 29 kg m^-2^. For logistical reasons they were combined into one group, due to the small number of subjects in each group and also the fact that four obese subjects had a borderline overweight/obese class I BMI (30–31 kg m^-2^). VAS/V_I_ intercept and slope data were tested and showed no significant differences between these overweight and obese subjects. Therefore, it was expected that this combination would have no material effect on the results.

It has been recommended that patients with respiratory disease should attempt to keep their BMI in the normal range (29), and our study has shown that this recommendation, with regard to the symptoms of dyspnoea and lung function, should be extended to healthy subjects. Additionally, previous work has shown that men with an abnormal BMI are at an increased risk of developing COPD (8), were associated with a higher prevalence of asthma (9), and in asthma would have a greater decline in FEV_1_ (30). We strongly agree with a suggestion that future clinical breathlessness studies should consider data stratification by BMI (10).

In conclusion, this study has shown that UW and OW healthy subjects showed greater levels of dyspnoea during exercise compared to NW subjects. Higher levels of dyspnoea could possibly result from a decrease in RMS. The reason for the greater dyspnoea of OW subjects could be an increase in the work of breathing. Implications from this study are that UW and OW subjects might be more susceptible to increased dyspnoea if they develop lung disease and, secondly, that caution is needed in clinical or mechanistic trials using subjects with abnormal body mass indices or body fat and varied levels of physical activity.
